# Bromide of Potassium

**Published:** 1873-07

**Authors:** 


### BROMIDE OF POTASSIUM.

We have received the following as a part of a private letter from our friend, Dr. Geo. D. Gray, of LaGrange, Lee county, Arkansas, which we regard as worthy of attention:

"I would like to call the attention of the profession to the use of bromide of potassium in the sick stomach and vomiting of bilious remittent fever. Yesterday I was called to a female aged thirty-five—has never been married. I found her with high fever, hot skin, pulse 120, tongue heavily coated, and dry, also with great thirst. The patient had taken several doses of the mild chloride of mercury, which had acted several times on her bowels, her discharges being of a dark, bilious looking character. Her vomiting was incessant. I first gave her ten grains subnitrate bismuth, and applied a blister to the epigastrium. That having failed, oxalate of cerium, creosote, the hypodermic injection of morphia was used, and nearly every other remedy I could think of, which afforded no relief whatever. The patient then being nearly exhausted, I finally gave her twenty grains of the bromide of potassium, and in less than ten minutes the patient was asleep. I would state that it had been used by me before, and my father (who thinks there is no other remedy like it in such cases) induced me to try it in this case, my own confidence in it not being so great before, as I could not account for its therapeutic action."

